# Gale G. Hannigan (1950–2024)

**DOI:** 10.5195/jmla.2026.2430

**Published:** 2026-01-01

**Authors:** Janis F. Brown

**Affiliations:** 1 jbrown@usc.edu, Associate University Librarian Emerita, Norris Medical Library, University of Southern California, Los Angeles, CA

**Figure:**
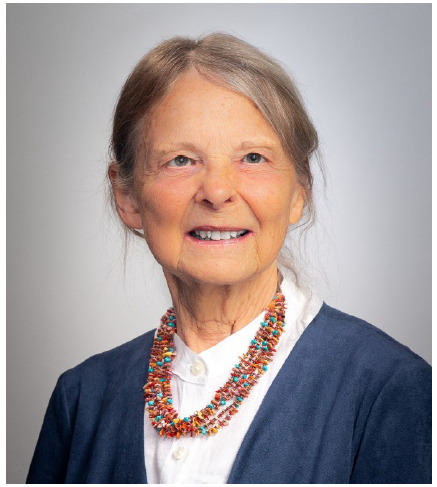


Gale Gabrielle Hannigan passed away on June 27, 2024, in Alburquerque, NM. Gale was born on October 9, 1950, at Flower Fifth Avenue Hospital in New York City. Gale enjoyed an idyllic childhood in Middletown, New Jersey, attending local schools during the winter and spending every day at the beach in the summer. She excelled in school, including winning the Betty Crocker Award. For college she chose the University of California, Berkeley, in the midst of its tumultuous Freedom of Speech Movement in the late 1960s. She convinced her parents that she would be safe, would not get involved: she was going to study pre-med. As soon as she got to Berkeley she registered as a philosophy major.

While at UC Berkeley, Gale received a bachelor's degree with honors in philosophy and English and then a master's degree in library and information science. She moved to Texas where she held her first professional position at the Houston Academy of Medicine-Texas Medical Center Library (HAM-TMC) from 1977 – 1982. After moving to the Midwest, she became the Head, Learning Resources Center, at the University of Minnesota from 1982 until 1986, and then the Manager, Medical Library Services, at the Upjohn Company. She returned to Texas in 1990 to be the Manager, Education and Information Services, at Texas A&M Medical Sciences Library.

In 1996, she held a joint appointment with the Texas A&M College of Medicine as the Director, Informatics for Medical Education. Her years in Texas included being an adjunct lecturer at the University of Texas School of Library and Information Science and later as adjunct faculty at the University of North Texas School of Library and Information Sciences. Always the lifelong learner, in May 2000, Gale earned her PhD in Information Science from the University of North Texas.

In 2011, after retiring from Texas A&M, she moved to Alburquerque, New Mexico, a place she loved for the majestic Sandia Mountains, the people, and the culture. Despite having retired, Gale continued her career on a part-time basis at the University of New Mexico Health Sciences Library & Informatic Center (UNM HSLIC), as a visiting professor and then as a research librarian allowing her to pursue her love of medical libraries while helping others and stretching her mind until the end of her life.

Gale married Steve Bartoldt while they were both at HAM-TMC, Steve as a medical resident and Gale as a clinical librarian. In the years that followed due to their careers, they moved to be together geographically, even if not in the same city and became collaborators writing several articles together, until they both retired in Albuquerque.

An important life event was her diagnosis of breast cancer in the late 1990's. She dealt with the diagnosis and treatment by using both her sense of humor and her information skills while seeing medical information from another viewpoint, as she described in a 1998 article in American Libraries [1]. She declared “war on cancer” and used a fried egg to describe her radiation treatment, as she navigated her way through treatment utilizing the information she gathered and the resources available to her.

Gale has always been an early adopter and committed to sharing her expertise with others. Early in her career at HAM-TMC, she was at the forefront of clinical librarianship publishing articles in the inaugural issue of the Journal of Clinical Librarianship [2] and the Journal of Family Practice [3]. Education also has been a consistent theme in her career. She had a passion for working with library school and medical students, mentoring colleagues, tutoring in local children's reading programs, and teaching English grammar online to a young family in Iran.

Throughout her career, Gale has been active in MLA and played many leadership roles serving on national MLA committees such as the Books Panel and the Nominating Committee, as well as being elected as chair of several sections including the Educational Media and Technology Section and the Medical Library Education Section. She used her warm and casual personality, her inclusiveness, and her creative ideas to be an effective leader. She was committed to her profession and to the Association. She continued to engage with MLA after her “retirement.” In her role as co-chair of the task force reviewing competencies, she was instrumental in the development of the MLA 2017 Competencies for Lifelong Learning and Success [4]. As a fitting end to her long commitment to the profession, her final contribution was as a co-author to a key JMLA article [5] that was published posthumously.

Gale received many of the most prestigious MLA awards showing her value to the profession and her reputation within it, including the 1996 Estelle Brodman Award for Academic Medical Librarian of the Year, the 2011 Lucretia W. McClure Excellence in Education Award, and in 2023 she was elected as an MLA Fellow for her “sustained and outstanding contributions to health sciences librarianship and to the advancement of the purposes of MLA.” She was also recognized by her colleagues at the UNM HSLIC with an Exemplary Service award, and by the University of North Texas Department of Information Sciences with its Outstanding Alumni Award. UNM HSLIC Executive Director Melissa Rethlefsen shared in her library's “Farewell to Gale Hannigan [5]” a testament to Gale's value, “I cannot really remember a time when I didn't recognize her name as one of the greats in our field. But, it wasn't until I got to work with her that I truly understood how great she actually was." Gale was also recognized by her community including an acknowledgement by the Navajo Nation for her public health outreach to them.

Gale had a beautiful spirit. She was always kind and giving to others, and she often became fast friends with those whom she encountered. During Covid as a way to keep connections with others and to remind us that we still lived in a world of beauty, Gale created a mailing list to share images of nature. At her passing, her mailing list included 75 people and became the vehicle for her sister Kathy to share the sad news about Gale. The list then became a spontaneous online memorial for all to share their love for Gale and her impact on their lives. Gale's friends included people from all parts of her life, including former and current colleagues, students, fellow charitable organization volunteers, her real estate agent, her cleaning professional, her oncology and end-of-life teams, and many others. She was an important person in the lives of many who will miss her greatly.
